# Talus osteomyelitis by *Candida krusei* with multiple huge cystic lesions: a case report and review of literatures

**DOI:** 10.1186/s12891-022-05648-4

**Published:** 2022-07-19

**Authors:** Hyungtae Kim, Su-Young Bae

**Affiliations:** Department of Orthopaedic Surgery, Inje University Sanggye Paik Hospital, College of Medicine, Inje University, 1342, Dongil-Ro, Nowon-Gu, Seoul, 01757 Republic of Korea

**Keywords:** Talus, Osteomyelitis, *Candida krusei*, Surgical debridement, Case report

## Abstract

**Background:**

Osteomyelitis due to *Candida krusei* are extremely rare, given that only six cases have been reported, all of which are limited to the patients with immunocompromising risk factors. Here we report a case of *C. krusei* osteomyelitis in an immunocompetent patient, presenting with multiple huge cystic lesions of talus.

**Case presentation:**

A 66-year-old female presented with one year history of painful swelling of right ankle and a draining sinus around lateral malleolus. Five months and three months ago, she had undergone arthroscopic synovectomy and bursectomy which revealed no causative organism. Open bursectomy with sinus tract excision was performed and intravenous antibiotic was administered. Two year after the surgery, the patient revisited the clinic for recurrent painful swelling with pus drainage at the same location. Multiple huge cystic lesions with osteolysis and sclerotic rim of talus were found and *C. krusei* was isolated from tissue culture. The patient received surgical debridement and prolonged antifungal treatment comprising caspofungin and voriconazole.

**Conclusions:**

In this case, *C. krusei* infection showed atypically aggressive osteolysis shown as multiple huge cystic abscess. High index of suspicion is critical for early diagnosis and treatment to prevent such devastating results even in an immunocompetent patient.

## Background

Candida osteomyelitis, as a form of invasive candidiasis, clinically present with equivocal symptoms in insidious manner and minimal to moderate inflammatory marker response, often making the diagnosis difficult [1]. The vertebrae are the most commonly involved sites, followed by femora, ribs, sternum and humeri. In contrast, osteomyelitis of foot bones is limited to only 3% of all Candida osteomyelitis cases, as reported by Slenker et al. [2]. The organism spreads by predominantly hematogenous route, direct inoculation and, rarely, by contiguous manner, and *Candida albicans* was identified in 65% of cases, although non-*albicans* Candidal infection is increasingly reported [3]. Albeit intact neutrophil function is critical to defense against Candida infection, surprisingly, less than 20% of all cases were associated with significant immunosuppression such as neutropenia, organ/bone marrow transplantation, underlying hematologic malignancy and others. Meanwhile, history of spine or cardiothoracic surgery and broad-spectrum antibiotics usage were involved in 62 and 40%, respectively [1, 2]. Here we present a case of talus osteomyelitis by *Candida krusei* in a patient without apparent trauma history or immunocompromising factors including acquired immunodeficiency syndrome (AIDS), diabetes or malignancy. To our knowledge, there has been only 6 cases of osteomyelitis due to *C. krusei*, including bilateral polymicrobial osteomyelitis of feet reported by Kaldau et al., and this case was distinctive in that the patient has no significant immunosuppressive risk factors [4–9].

## Case presentation

**Fig. 1:**
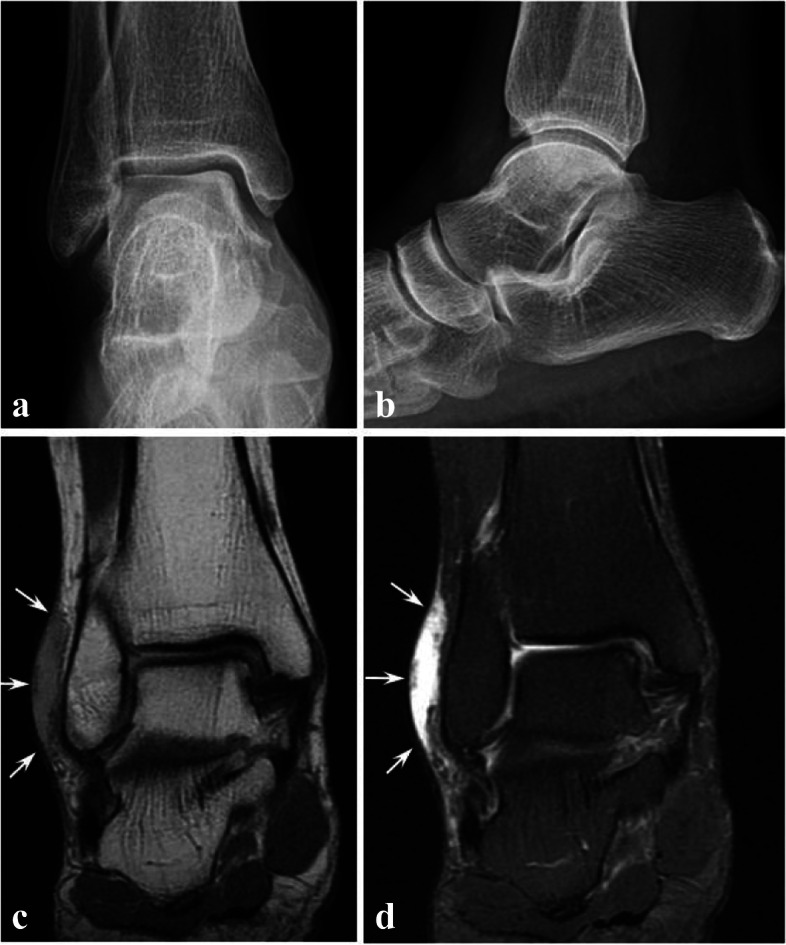
Initial plain radiographs and MRI from outside hospital. Plain radiographs (**a**, **b**) showing no osteolytic lesions around ankle joint. Coronal view of Proton density fat suppression image (**c**) and T2-weighted fat suppression image (**d**) showing lateral malleolar bursitis (white arrows)

**Fig. 2:**
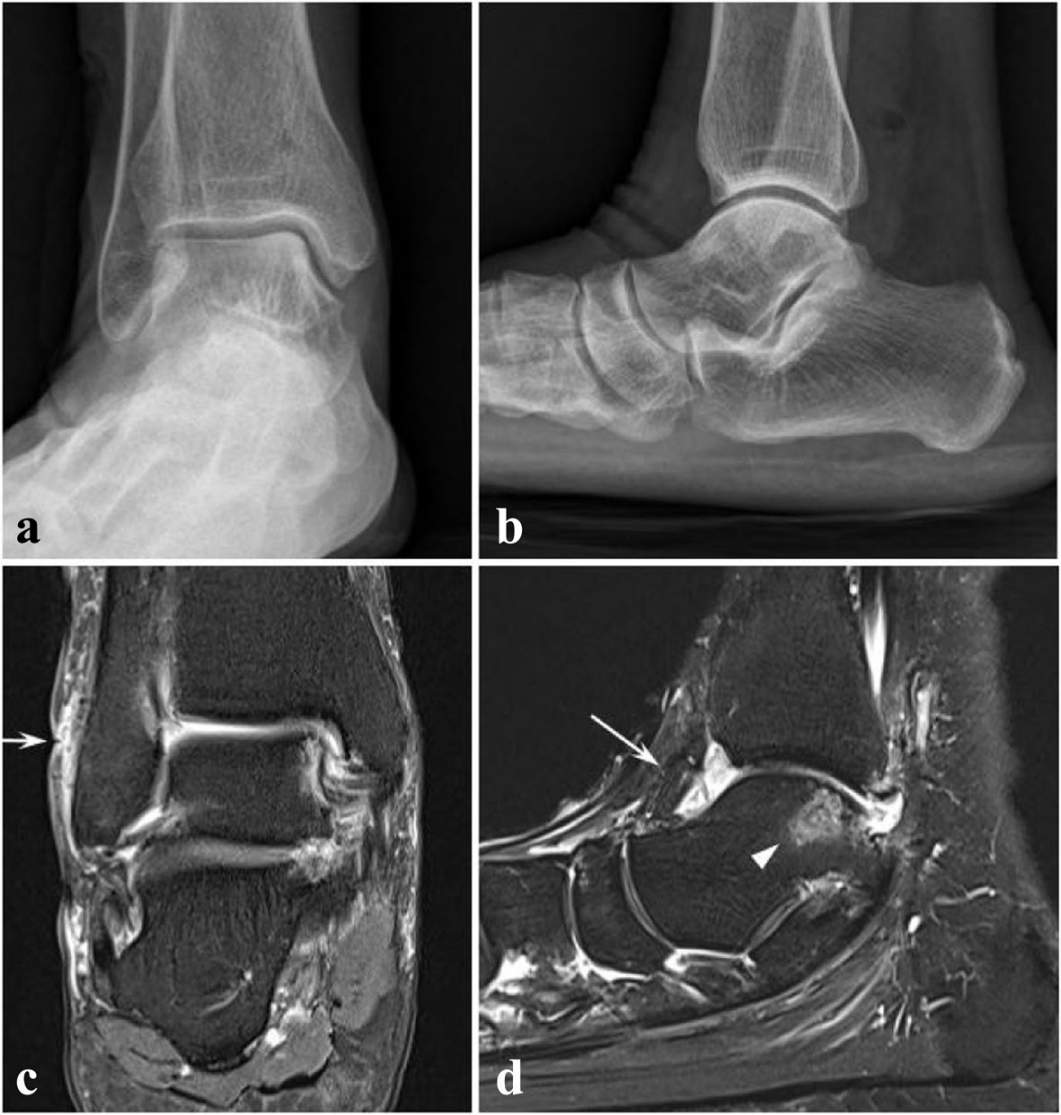
Plain radiographs and MRI of initial outpatient clinic visit. Plain radiographs (**a**, **b**) showing no significant changes from the initial images of outside hospital. Coronal view of proton density fat suppression image (**c**) showing thickened skin with focal defect over lateral malleolus (white arrow) with high signal intensity of subcutaneous layer, suggesting lateral malleolar bursitis with fistula. Sagittal view of T1-weighted double fat suppression images (**d**) showing focal bone marrow edema in talus (white arrow head) and synovial hypertrophy with effusion (white arrow) in right ankle joint, which was interpreted as nonspecific synovitis, with differential diagnosis of inflammatory arthritis, infective arthritis such as pyogenic or early tuberculous arthritis and reactive arthritis associated with ankle instability

A 66-year-old female visited the outpatient clinic with one year history of painful swelling of right ankle and a draining sinus around lateral malleolus. Her medical history was not significant except dyslipidemia and she had no apparent trauma before the onset of the symptoms. Five months and three months before the initial visit, she had undergone arthroscopic synovectomy and bursectomy in other hospital for chronic bursitis (Fig. 1), but no causative organism was revealed from the intraoperative tissue cultures. No limitation of range of motion or fever was observed at the initial presentation but the patient had intermittent pain with walking. Her laboratory tests showed no evidence of prominent infection, as white blood cell (WBC) count of 3700 /μL, Erythrocyte sedimentation rate (ESR) of 15 mm/hr., C-reactive protein (CRP) of < 0.3 mg/dL. No suspicious radiolucent lesion was observed on plain radiograph of her right ankle. (Fig. 2a, b) From her magnetic resonance image (MRI), high signal intensity in Proton density fat suppression images and T1-weighted double fat suppression images was found on subcutaneous tissue over lateral malleolus with intra-articular fluid effusion, synovial hypertrophy and localized bone edema around the ankle joint. (Fig. 2c, d) Chronic bursitis with sinus tract and nonspecific synovitis of ankle were diagnosed, with suggestion of differential diagnosis of inflammatory arthritis, septic arthritis, early tuberculous arthritis and reactive synovitis due to ankle instability.

Open bursectomy and sinus tract excision were performed, followed by 1st generation cephalosporin administration after the surgery. (Cefazedone 1 g BID [bis in die]) Bone biopsy was obtained from discolored distal fibular tip, which showed chronic inflammation and degeneration. Gram positive rods were identified in 1 out of 3 bacterial culture samples, one tissue culture sample and two swab cultures, and was interpreted as contamination as only one of the two swab samples showed gram positive bacilli, which seemed to be normal skin flora or contamination from surrounding environment according to the laboratory medicine department of the institution. Tuberculosis-polymerase chain reaction (TB-PCR) test was negative. The patient showed no fever postoperatively and CRP of < 0.3 mg/dL. 1st generation cephalosporin was administered intravenously until 2 weeks after the surgery and changed to oral agent for 1 more week. The patient was followed until 22 weeks postoperatively without any symptoms or signs of recurrence.

**Fig. 3:**
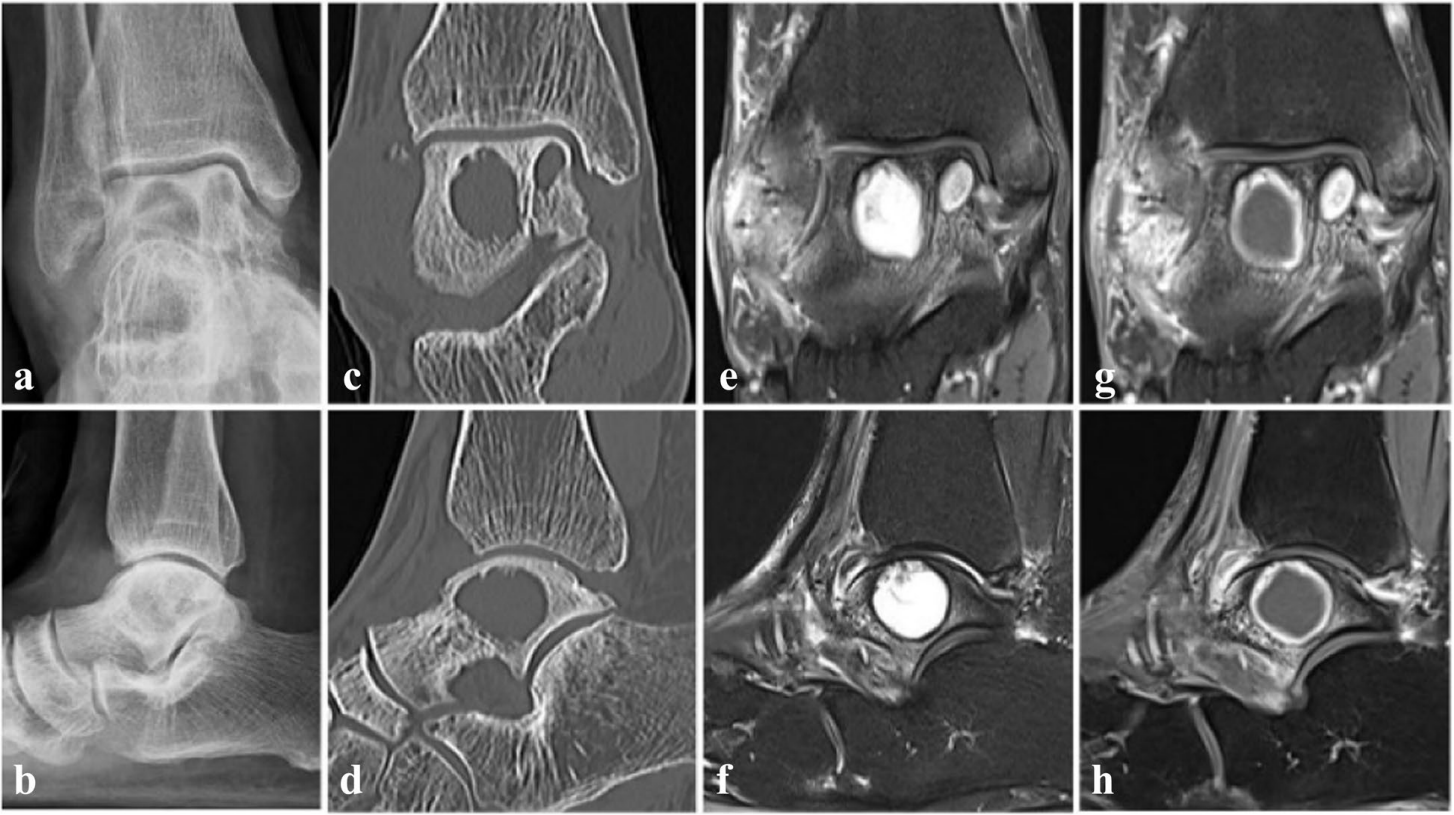
Plain radiograph, CT and MRI after recurrence**.** Plain radiographs in anteroposterior and lateral view (**a**, **b**) showing multiple huge osteolytic lesions of talus. CT scan in coronal and sagittal view (**c**, **d**) showing large osteolytic lesions with sclerotic rim yet without talar roof collapse. Proton density and T2-weighted images with fat suppression view (**e**, **f**) revealed cystic abscess of talus with sclerotic rim and surrounding bone marrow edema. Wall enhancement of the cystic lesions with Gadolinium contrast was observed (**g**, **h**)

**Fig. 4:**
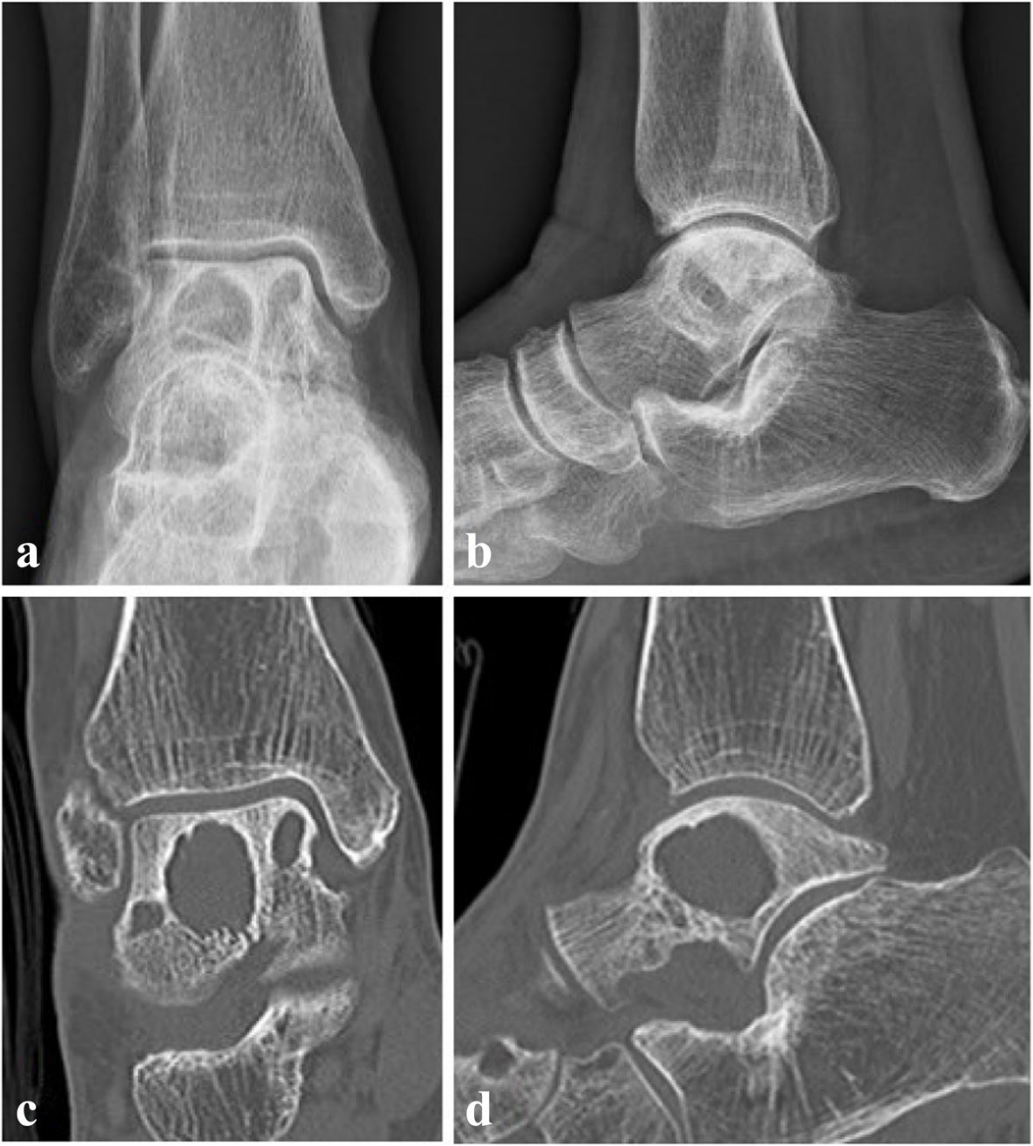
Plain radiographs and CT images of postoperative 10 months. Plain radiographs (**a**, **b**) and CT (**c**, **d**) showing stationary cystic lesions of talus without definitive progression

**Fig. 5:**
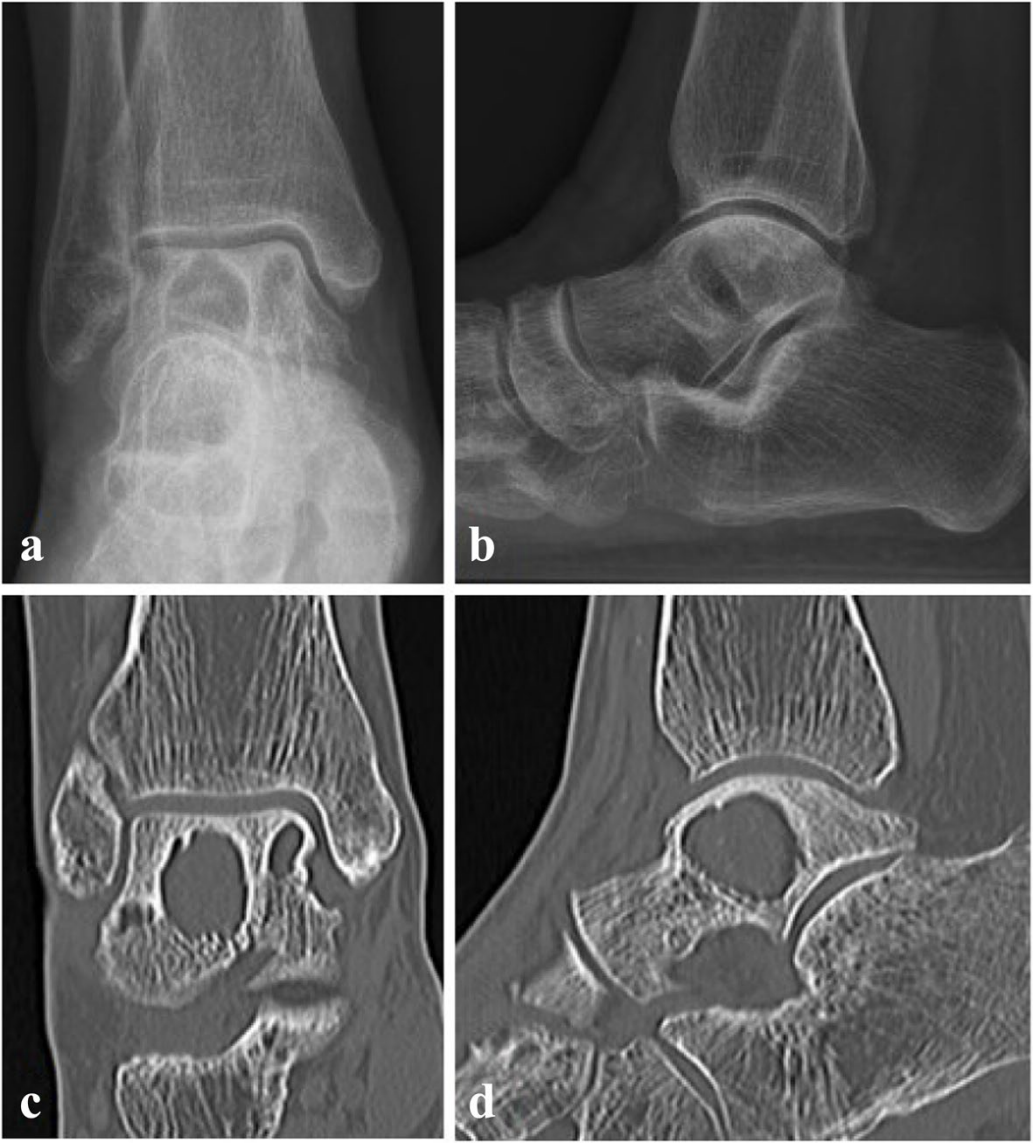
Plain radiographs and CT images of postoperative 16 months. Plain radiographs (**a**, **b**) and CT (**c**, **d**) showing slightly thickened cystic wall without any evidence of talar collapse

The patient revisited the clinic two years later from the surgery for recurred painful swelling and pus drainage. Plain radiograph, computed tomography (CT) and MRI were taken and multiple huge cystic abscess surrounded by sclerotic rim and osteomyelitis of talus and medial malleolus were identified. (Fig. 3) Lateral malleolar bursectomy, debridement and drainage of talus abscess were done and intraoperative frozen biopsy obtained from periarticular soft tissue and bursae showed low grade infectious soft tissue. Permanent tissue biopsy was interpreted as chronic osteomyelitis with fibrinoid exudate, fibrosis and necrosis with abscess and foreign body reaction. Anti-nuclear antibody, anti-cyclic citrullinated peptide antibody and rheumatoid factor were tested to exclude autoimmune disease and other inflammatory arthritis, which turned out to be negative for all three markers. Bacterial culture, acid fast bacteria stain, TB-PCR, potassium hydroxide (KOH) mount and fungal culture were obtained and *C. krusei* was identified 1 out of 2 fungal culture samples. Antifungal drug sensitivity and resistance test were requested and the pathogen confirmed to be sensitive to amphotericin B, micafungin and voriconazole, intermediate for caspofungin, and resistant to fluconazole and flucytosine. Even though in vitro susceptibility against caspofungin was intermediate, caspofungin was recommended by the infectious disease specialist of the hospital according to the sensitivity test result, considering the potential toxicity of amphotericin B to which the pathogen was more susceptible. After two weeks of intravenous caspofungin 50 mg QD [quaque die] administration, the patient was discharged with patellar tendon bearing cast and oral voriconazole 200 mg BID for 6-month period, which was tolerable with only minor adverse effects like dry mouth, reported by the patient. The patient had regular liver function tests to monitor hepatotoxicity of voriconazole during antifungal administration at local clinic, all of which were within normal limits according to the patient. Her last outpatient clinic visit after one year and four months from the last surgery was uneventful with no clinical symptom recurrence. Plain radiographs and CT scan showed stationary cystic lesions of talus without evident talar roof collapse, and when compared to the radiographic studies of 10 months after the last surgery (Fig. 4), slightly thickened cortical lining of the talar cysts was observed. (Fig. 5) Possibility of bone graft to the void was explained to the patient for prevention of potential talar collapse, but she refused to undergo another surgery as she had no clinical symptoms or functional impairments.

## Discussion and conclusions

Candida osteomyelitis is an uncommon disease, occurring mostly after systemic candidemia or spine and cardiothoracic surgery, and the cases affecting extremities are even more rarely reported [1, 2]. Nonetheless, infectious conditions by atypical pathogens such as *Mycobacterium tuberculosis* and fungi should be differentiated in patients presenting with chronic, insidious disease course and muted inflammatory marker response [10].

Clinically, fungal infection is not highly considered in osteomyelitis of extremities when the patient has no immunocompromising risk factors or prominent trauma history such as open fracture. Gamaletsou et al. reported in a case series of 207 Candida osteomyelitis that severe immunosuppression was not found in majority of the cases, which is consistent with the report by Slenker et al. In the literature, the most commonly associated factor was prior abdominal, cardiothoracic and spinal surgeries in 62%, followed by the prior use of broad-spectrum antibiotics (40%), intravenous drug abuse (19%), central venous catheter insertion (19%) and, notably, immunosuppression in only 17% of cases [1, 2]. In addition, Cheon et al reported a case of fungal arthritis and osteomyelitis of ankle after repetitive intra-articular steroid injections in a diabetic patient, which reminds us the necessity of detailed history taking about iatrogenic factors and previous surgeries [11].

Among the Candida species, *Candida albicans* is the most common pathogen, however, recently non-*albicans* Candida infections are increasing [3]. Especially, *C. krusei* has intrinsic resistance against fluconazole and low susceptibility to amphotericin B and flucytosine, at the same time, it develops acquired resistance to other antifungals enhanced by its ability to form biofilm [3, 12, 13]. Moreover, the yeast has lowest 90-day survival rate when compared to other systemic Candida species infections [14].

Radiographic findings of osteomyelitis by Candida are nonspecific and similar to those of pyogenic osteomyelitis, such as synovial thickening, joint effusion, articular cartilage destruction, subchondral bone loss and bone marrow edema, which makes the early diagnosis more difficult [15]. Initially, MRI of the patients disclosed nonspecific changes including bursitis and early inflammatory or atypical infectious arthritis such as tuberculosis. However, the MRI obtained when the disease had recurrence 2 year after the surgery showed large osteolytic lesions around ankle joint and within the talus, which could have led to the collapse of talar roof.

The mainstay of treatment for Candida osteomyelitis is surgical debridement and antifungal treatments, as suggested by Infectious Diseases Society of America [16]. In extreme cases, persistent infection can lead to limb amputation even after the surgery and long-term antifungal administration [9]. It is difficult to diagnose at the beginning of the infection, considering its slow and less aggressive natural history, and relapsing is commonly observed even after complete or partial response, mainly due to premature discontinuation of antifungal therapy. Gamaletsou et al. reported that 32 and 27% of cases had experienced relapse who ultimately achieved complete and partial resolution, respectively [1]. Thus, a delay in the diagnosis and treatment can lead to progression of the infection, causing critical outcomes including limb amputation and significant bone destruction. In this case, 2 weeks of caspofungin and 6 months of voriconazole therapy ended up with no recurrence until 16 months from surgical intervention.

Up to date, six cases of *C. krusei* osteomyelitis have been reported. (Table 1) Three cases of spondylodiscitis and three cases affecting sternum, tibia and foot, respectively, can be found in the literature, all of which are limited to the patients with significant immunosuppression such as chemotherapy in leukemia, bone marrow transplantation and broad spectrum antibiotics treatment [4–9]. This is the first case of *C. krusei* osteomyelitis of foot bones in an immunocompetent patient. The initial onset of the symptoms was several months before the first surgical intervention in the local hospital and she had no definitive trauma history or environmental exposure, which makes the route of the infection unclear. The mild, relapsing characteristic of the infection without prominent laboratory results was the only clue for the early diagnosis. As mentioned in previous articles, high index of suspicion for fungal infection should be maintained in a patient presented with ambiguous symptoms of infection sustained for a relatively long period. Jarren et al. spoke against routine acquisition of fungal and acid fast bacteria culture samples in musculoskeletal infection of pediatric population, unless there were immunocompromising risk factors, due to low likelihood of these atypical infections [17].

**Table 1:** Summary of previously reported *Candida krusei* cases

Reference	Petrikkos et al., 2001 [4]	Pemán et al., 2006 [5]	Schilling et al., 2008 [6]	Overgaauw et al., 2020 [7]	Mayer et al., 2013 [8]	Kaldau et al., 2012 [9]
Sex/Age	F/75	M/62	M/58	M/78	F/47	M/60
Risk factors	Sternotomy for CABG	AML, CTx	AML, CTx, AB	AML, CTx	AML, immunosuppression after alloSCT	AB for gallstone pancreatitis
Candidemia	No	Yes	Yes	Yes	No	No
Location	Sternum	Spine	Spine	Spine	Tibia	Multiple foot bone including talus and calcaneus
Diagnostic method	Pus culture from sternotomy site	Blood culture, CT-guided fine needle biopsy	Blood culture, bronchial lavage	Abscess aspiration culture, bone biopsy culture	Joint fluid culture from aspiration	Tissue culture from calcaneus
Antifungal treatment	Itraconazole for 6 months	Caspofungin, voriconazole for 6 w	Caspofungin, posaconazole for 1 y	Anidulafungin, voriconazole for 6 m	Anidulafungin, voriconazole for 3 m	Liposomal amphotericin B for 4 m
Surgical intervention	No	No	Yes	Yes	Yes	Yes

CABG: coronary artery bypass graft surgery; AML: acute myeloid leukemia; CTx: chemotherapy; AB: antibiotics; alloSCT: allogenic peripheral blood stem cells transplantation; M: male; F: female; w: weeks; m: months; y: years

Nevertheless, considering the fact that only limited number of Candida osteomyelitis cases was associated with significant immunosuppression and the consequences when a clinician missed such tenacious infection, it would be beneficial to differentiate fungal infections even in the immunocompetent patients. At least, when the patient presents with chronic, vague and recurring symptoms, this rare infection should be on the physician’s list of differential diagnoses. Further studies and reports about the characteristics and treatment outcomes of this rare fungal infection is needed considering its rising incidence and virulence associated with intrinsic and acquired antifungal resistance.

## Data Availability

All data generated or analysed during this study are included in this published article and its supplementary information files.

## Abbreviations

AIDS: Acquired immunodeficiency syndrome
WBC: White blood cell
ESR: Erythrocyte sedimentation
CRP: C-reactive protein
MRI: Magnetic resonance imaging
CT: Computed tomography
TB-PCR: Tuberculosis-polymerase chain reaction
KOH: Potassium hydroxide
BID: bis in die
QD: quaque die
*C. albicans*: *Candida albicans*
*C. krusei*: *Candida krusei*

## References

[1] Gamaletsou MN, Kontoyiannis DP, Sipsas NV, Moriyama B, Alexander E, Roilides E (2012). Candida osteomyelitis: analysis of 207 pediatric and adult cases (1970-2011). Clin Infect Dis.

[2] Slenker AK, Keith SW, Horn DL (2012). Two hundred and eleven cases of Candida osteomyelitis: 17 case reports and a review of the literature. Diagn Microbiol Infect Dis.

[3] Jamiu AT, Albertyn J, Sebolai O, Gcilitshana O, Pohl CH (2021). Inhibitory effect of polyunsaturated fatty acids alone or in combination with fluconazole on Candida krusei biofilms in vitro and in Caenorhabditis elegans. Med Mycol.

[4] Petrikkos G, Skiada A, Sabatakou H, Antoniadou A, Dosios T, Giamarellou H (2001). Case report. Successful treatment of two cases of post-surgical sternal osteomyelitis, due to Candida krusei and Candida albicans, respectively, with high doses of triazoles (fluconazole, itraconazole). Mycoses..

[5] Pemán J, Jarque I, Bosch M, Cantón E, Salavert M, de Llanos R (2006). Spondylodiscitis caused by *Candida krusei* : case report and susceptibility patterns. J Clin Microbiol.

[6] Schilling A, Seibold M, Mansmann V, Gleissner B (2008). Successfully treated *Candida krusei* infection of the lumbar spine with combined caspofungin/posaconazole therapy. Med Mycol.

[7] Overgaauw AJC, de Leeuw DC, Stoof SP, van Dijk K, Bot JCJ, Hendriks EJ (2020). Case report: Candida krusei spondylitis in an immunocompromised patient. BMC Infect Dis.

[8] Mayer K, Kapelle M, Kaeferstein A, Weßling M, Bekeredjian-Ding I, Leutner C (2013). Successful management of Candida krusei monoarthritis after Allo-SCT. Bone Marrow Transplant.

[9] Kaldau NC, Brorson S, Jensen P-E, Schultz C, Arpi M (2012). Bilateral polymicrobial osteomyelitis with Candida tropicalis and Candida krusei: a case report and an updated literature review. Int J Infect Dis.

[10] Yingling JM, Sun L, Yoon R, Liporace F (2017). A rare case of Candida parapsilosis osteomyelitis: a literature review and proposed treatment algorithm. Patient Saf Surg.

[11] Cheon SM, Park HY, Moon JY, Yoon JH, Moon W, Hong SM (2005). A case of Candida Parapsilosis ankle arthritis after intra-articular steroid injection. Infect Chemother.

[12] Jamiu AT, Albertyn J, Sebolai OM, Pohl CH (2021). Update on Candida krusei, a potential multidrug-resistant pathogen. Med Mycol.

[13] Multani A, Subramanian AK, Liu AY (2019). Successful eradication of chronic symptomatic Candida krusei urinary tract infection with increased dose micafungin in a liver and kidney transplant recipient: case report and review of the literature. Transpl Infect Dis Off J Transplant Soc.

[14] van Haren MHI, de Groot T, Spruijtenburg B, Jain K, Chowdhary A, Meis JF (2022). Development of a multiplex PCR short tandem repeat typing scheme for Candida krusei. J Clin Microbiol.

[15] Sung J, Chun KA (2011). Candida Parapsilosis arthritis involving the ankle in a diabetes patient: a case report. J Korean Soc Radiol.

[16] Pappas PG, Kauffman CA, Andes D, Benjamin DK, Calandra TF, Edwards JE (2009). Clinical practice guidelines for the management of candidiasis: 2009 update by the Infectious Diseases Society of America. Clin Infect Dis Off Publ Infect Dis Soc Am.

[17] Section J, Gibbons SD, Barton T, Greenberg DE, Jo C-H, Copley LAB (2015). Microbiological culture methods for pediatric musculoskeletal infection: a guideline for optimal use. J Bone Jt Surg.

